# Supplementary material for: Classification of glucose-level in deionized water using machine learning models and data pre-processing technique
Source: PLoS One. 2024 Dec 5;19(12):e0311482. doi: 10.1371/journal.pone.0311482 (PMC11620423; doi:10.1371/journal.pone.0311482)
Supplement: Attachment — Submitted filename: PLOS ONE REVIEW - Classification of glucose-level in deionized water using machine learning models and.docx [file pone.0311482.s001.docx]

Classification of glucose-level in deionized water using machine learning models and

data pre-processing technique

The overall quality of the paper is poor. This manuscript was not well written and suggest a rejection of the manuscript. The line in the whole document is not numbered

1. I suggest the authors get editing help from someone with full professional proficiency in English. There are improper or ambiguous English usages. The writing of the paper needs to be polished.
2. Abstract is not well written.
3. The introduction section is not well organized.
4. Methodology is poorly written.
