# Supplementary material for: Classification of glucose-level in deionized water using machine learning models and data pre-processing technique
Source: PLoS One. 2024 Dec 5;19(12):e0311482. doi: 10.1371/journal.pone.0311482 (PMC11620423; doi:10.1371/journal.pone.0311482)
Supplement: Attachment — Submitted filename: Classification of glucose.docx [file pone.0311482.s002.docx]

The authors described the classification of glucose-level in deionized water using machine learning models coupled with Raman spectroscopy. Before publication can be considered, the authors need to address the following issues:

1. Authors should specify and comment (in abstract) on their main findings

2. The authors indicated measuring the analyte at 10 different concentrations but do not mention how many times they measure each concentration, especially the best peak.

3. How about signal reproducibility of the proposed method?

4. The authors should highlight the limitations and strengths of their proposed method

5. Change Fig 1a to Fig 2a and Fig 1b to Fig 2b

6. The authors used lots of abbreviations that should be properly defined in the text
