# Supplementary material for: Classification of glucose-level in deionized water using machine learning models and data pre-processing technique
Source: PLoS One. 2024 Dec 5;19(12):e0311482. doi: 10.1371/journal.pone.0311482 (PMC11620423; doi:10.1371/journal.pone.0311482)
Supplement: Attachment — Submitted filename: PONE-D-23-21128R1-.docx [file pone.0311482.s004.docx]

Authors are encouraged to refine the grammar and syntax of the manuscript. The numbering of the various sub-headings must also re-looked at
